# Comprehensive analysis of codon bias in 13 *Ganoderma* mitochondrial genomes

**DOI:** 10.3389/fmicb.2023.1170790

**Published:** 2023-05-04

**Authors:** Peng Wu, Wenqi Xiao, Yingyong Luo, Zhuang Xiong, Xiaodie Chen, Jing He, Ajia Sha, Mingying Gui, Qiang Li

**Affiliations:** ^1^Yunnan Plateau Characteristic Agricultural Industry Research Institute, Yunnan Agricultural University, Kunming, Yunnan, China; ^2^Key Laboratory of Coarse Cereal Processing, Ministry of Agriculture and Rural Affairs, Sichuan Engineering and Technology Research Center of Coarse Cereal Industrialization, School of Food and Biological Engineering, Chengdu University, Chengdu, Sichuan, China

**Keywords:** codon usage, mitochondrial genome, genetics, natural selection, fungi

## Abstract

**Introduction:**

Codon usage bias is a prevalent phenomenon observed across various species and genes. However, the specific attributes of codon usage in the mitochondrial genome of *Ganoderma* species remain unknown.

**Methods:**

In this study, we investigated the codon bias of 12 mitochondrial core protein-coding genes (PCGs) in 9 *Ganoderma* species, including 13 *Ganoderma* strains.

**Results:**

The codons of all *Ganoderma* strains showed a preference for ending in A/T. Additionally, correlations between codon base composition and the codon adaptation index (CAI), codon bias index (CBI) and frequency of optimal codons (FOP) were identified, demonstrating the impact of base composition on codon bias. Various base bias indicators were found to vary between or within *Ganoderma* strains, including GC3s, the CAI, the CBI, and the FOP. The results also revealed that the mitochondrial core PCGs of *Ganoderma* have an average effective number of codons (ENC) lower than 35, indicating strong bias toward certain codons. Evidence from neutrality plot and PR2-bias plot analysis indicates that natural selection is a major factor affecting codon bias in *Ganoderma*. Additionally, 11 to 22 optimal codons (ΔRSCU>0.08 and RSCU>1) were identified in 13 *Ganoderma* strains, with GCA, AUC, and UUC being the most widely used optimal codons in *Ganoderma*. By analyzing the combined mitochondrial sequences and relative synonymous codon usage (RSCU) values, the genetic relationships between or within *Ganoderma* strains were determined, indicating variations between them. Nevertheless, RSCU-based analysis illustrated the intra- and interspecies relationships of certain *Ganoderma* species.

**Discussion:**

This study deepens our insight into the synonymous codon usage characteristics, genetics, and evolution of this important fungal group.

## Introduction

The genetic data present in the DNA of organic material is converted into a sequence of 20 amino acids through the mechanisms of transcription and translation (Iriarte et al., 2021; Parvathy et al., 2022). The 64 triplet codon arrangements found in DNA are responsible for encoding 20 standard amino acids, with 61 triplets dedicated to this purpose. Eighteen of the amino acids are represented by multiple codons, while tryptophan and methionine are typically encoded by a single codon. The degeneracy of the genetic code allows the same amino acid to be represented by synonymous codons or divergent codons (Krasovec and Filatov, 2019; Pepe and Keersmaecker, 2020). Synonymous codons are not usually used randomly or with equal probability; rather, some codons are more frequently utilized than others to encode amino acids. This phenomenon is known as codon usage bias (CUB) (Biro, 2008; Behura and Severson, 2013). Synonymous codons frequently appear with different frequencies in different genes, different organisms, or even the same gene from different species (Akashi, 1997; Choudhury et al., 2018; Camiolo et al., 2019). Substituting one codon for another synonymous codon will not result in any alteration of the primary sequence of amino acids or peptides, which is primarily caused by mutations in the gene coding region, particularly mutations in the second or third nucleotides of the codon (De Mandal et al., 2020; Bailey et al., 2021). Evolutionary processes can lead to variability in synonymous codons due to a synonymous mutation or ‘silent mutation’, which does not have any impact on functionality (Deng et al., 2020; De Oliveira et al., 2021). It can be concluded that codon usage bias is the consequence of mutation patterns that are unevenly distributed. As some codons are more prone to mutation than others, selection is able to maintain this bias (Rao et al., 2011; Trotta, 2013). GC heterogeneity and gBGC can lead to codon usage bias, which is a consequence of local recombination rate-based codon usage (Palidwor et al., 2010; Oldfield et al., 2020). Mutations, natural selection, and genetic drift are all factors that contribute to the development of synonymous codons and thus have a considerable effect on genome evolution (Li et al., 2018; Li B. et al., 2022). It has been proposed that interspecific differences in codon usage can be explained by a mutation mechanism in which codon bias is caused by the rate of repair of nucleotide bias or point mutations (LaBella et al., 2019; Lamolle et al., 2022; Li et al., 2022c, 2023b). Natural selection theory postulates that synonymous mutations that have an effect on an organism’s ability to adapt will be either favored or disfavored over the course of evolution, resulting in changes in codon usage in genomes or genes (Dhindsa et al., 2020; Cope and Shah, 2022).

Codon bias can have a significant impact on a variety of cellular processes and characteristics, such as transcription, translation efficiency and accuracy, mRNA stability, and protein expression, structure, function, and folding during cotranslation (Chaney and Clark, 2015; Fages-Lartaud et al., 2022). Codon bias has an influence on transcription due to its effect on chromatin structure and mRNA folding, which in turn affects translation efficiency by influencing the rate of translation elongation (Hia et al., 2019; Hia and Takeuchi, 2021). The genome has adapted to transcription and translation procedures through the development of codon bias. This increases the effectiveness of studies on the molecular evolution of genes by allowing selection of genes that do not alter amino acids (Yao et al., 2019; Yu et al., 2021). Furthermore, codon bias analysis can reveal related organisms (Li et al., 2023a), as they often use similar codons (Prabha et al., 2017; Rahman et al., 2022). By utilizing genetic engineering and recombinant DNA technology, the expression of heterologous genes can be enhanced by optimizing the codons of the genes that code for the most highly expressed proteins (López et al., 2020; Liu et al., 2021; Bao et al., 2023). The advancement of high-throughput sequencing technology has made codon bias analysis an indispensable tool for exploring species evolution, environmental adaptation, and genetics (Lal et al., 2016; Masłowska-Górnicz et al., 2022; Wu et al., 2023). However, the genetic features of codon bias in large, higher fungi are not yet fully understood (Jiang et al., 2017).

Eukaryotes rely on the mitochondrial genome, which is often referred to as the ‘second genome’, for their growth, development, and ability to adjust to the environment (Latorre-Pellicer et al., 2016). Fungi possess a set of 15 core protein-coding genes (PCGs), namely, *atp6*, *atp8*, *atp9*, *cob*, *cox1*, *cox2*, *cox3*, *nad1*, *nad2*, *nad3*, *nad4*, *nad4*L, *nad5*, *nad6*, and *rps3* (Li et al., 2020a, 2022a,b). Variations in the mitochondrial genome are essential for the stability, resilience, and adaptability of eukaryotic cells, in addition to their development (Hayashi et al., 2016; Chen et al., 2020; Cai et al., 2021). *Ganoderma* is a genus of fungi that is part of the Ganodermataceae family, a group of fungi with a wide variety of species (Kwon et al., 2016; Sun et al., 2022a,c). It is believed that the genus *Ganoderma* encompasses more than 250 distinct species (Wang et al., 2012; Kües et al., 2015; Jargalmaa et al., 2017; Sun et al., 2022c). At present, the genomics and cultivation of *Ganoderma* genus are receiving increasing attention, promoting the development and utilization of *Ganoderma* resources (Si et al., 2021; Sun et al., 2022b). It has been observed that some species from the *Ganoderma* genus can be detrimental to woody plants, such as *G*. *boninense* (Dhillon et al., 2021), *G. steyaertanum* (Hidayati et al., 2014), *G. mastoporum* (Page et al., 2018), and *G. philippii* (Francis et al., 2014). Numerous *Ganoderma* species are renowned for their medicinal properties in traditional Asian medicine (Paterson, 2006; Bishop et al., 2015; Zhu et al., 2015). Some *Ganoderma* species, including *G. lucidum*, *G. leucocontextum*, *G. sinense*, and *G. lingzhi*, have been found to have a variety of beneficial ingredients that are of great economic and medical value (Chan et al., 2015; Chien et al., 2015; Gao et al., 2020; Zheng et al., 2020). The bioactive components of *Ganoderma* have drawn increasing attention from the Western medical and wellness industries, leading to a multibillion dollar market value in 2017 (Jargalmaa et al., 2017). To date, the codon usage, genetic properties, and evolutionary development of the mitochondrial core proteins in *Ganoderma* have not been explored.

This study investigated the utilization of synonymous codons of mitochondrial core PCGs between and within 13 *Ganoderma* strains, including *G. applanatum*, *G. leucocontextum*, *G. tsugae*, *G. sinense*, *G. subamboinense* s118, *G. calidophilum*, *G. meredithae*, *G. lucidum* KC763799, *G. lucidum* s26, *G. lucidum* s37, *G. lingzhi* s62, *G. lingzhi* s74, and *G. lingzhi* s8. Utilizing relative synonymous codon usage (RSCU) data, we deduced the phylogenetic relationship of different *Ganoderma* strains and compared it with the phylogenetic relationship established through mitochondrial genome sequence inference. This is the first study to investigate the synonymous codon usage patterns of this important group of edible and medicinal fungi, which will contribute to a better understanding of the evolution, genetics, and species differentiation of *Ganoderma* and other related species.

## Materials and methods

### Sequence processing

To date, 13 full *Ganoderma* mitochondrial genomes have been added to the NCBI database, 9 of which were reported in our prior research (Li et al., 2019b, 2022d). We first downloaded the *G. applanatum*, *G. leucocontextum*, *G. tsugae*, *G. calidophilum*, *G. subamboinense*, *G. meredithae*, *G. sinense*, *G. lucidum*, *G. lucidum* s26, *G. lucidum* s37, *G. lingzhi* s62, *G. lingzhi* s74, and *G. lingzhi* s8 mitochondrial genomes from the NCBI database under the accession numbers KR109212, MH252534, MH252533, MH252535, MW752412, KP410262, KF673550, KC763799, MH252532, MW752414, MW752415, MW752413, and MH252531, respectively (Li et al., 2013; Wang et al., 2016a,b; Utomo et al., 2019; Meng et al., 2022). We then obtained the core protein-coding sequence in the mitochondrial genomes of 13 *Ganoderma* strains. Core protein-coding genes with a sequence length of less than 300 bp were excluded from the subsequent analysis (Bu et al., 2021). Consequently, we identified 12 core protein-coding genes in each *Ganoderma* strain for further study, comprising *atp6*, *cob*, *cox1*, *cox2*, *cox3*, *nad1*, *nad2*, *nad3*, *nad4*, *nad5*, *nad6*, and *rps3*.

### Codon usage indices

The GC3s metric is utilized to quantify the number of codons with guanine and cytosine at the third synonymous position, with the exception of Met, Trp, and stop codons (Huo et al., 2021; Xu et al., 2021). The codon adaptation index (CAI) is a numerical value used to gage the propensity for codons to be utilized in genes that have been expressed at high levels (Sharp and Li, 1987). The CAI is a measure that indicates the frequency of synonymous codon usage and is expressed as a numerical value from 0 to 1.0, with higher values indicating a higher frequency. The codon bias index (CBI) is a measure used to assess gene expression based on the relative abundance of optimal codons in a gene. The frequency of optimal codons (FOP) is calculated by dividing the number of optimal codons by the total number of synonymous codons in the gene. The effective number of codons (ENC) is a metric for evaluating the number of codons present in a gene, which can range from 20 to 61. A score of 20 implies that only one codon is allocated for each amino acid, whereas a result of 61 implies that each codon is employed with equal frequency. The ENC value is a measure of codon usage preference; a low value (below 35) signifies a strong preference, while a higher value (above 35) indicates a weaker preference. The relative synonymous codon usage (RSCU) is calculated by dividing the probability of the same codons encoding the same amino acid. Codon bias is deemed to be positive when the RSCU value is above 1 and negative when the value is below 1. The general average hydropathicity (GRAVY) value is calculated by adding up the hydropathy values of all the amino acids in the polymerase gene sequences and then multiplying the sum by the number of residues in the gene sequences. This value ranges from −2 to 2, with positive values indicating hydrophobic proteins and negative values indicating hydrophilic proteins. The aromaticity (AROMO) value is a metric of the frequency of aromatic amino acids (Phe, Tyr, and Trp). GRAVY and AROMO are also metrics of amino acid usage, and any changes in the amino acid composition will have an impact on the results of codon usage. All codon usage indicators can be derived through the use of CodonW1.4.2 (Peden, 1999) or the CAIcal server (Puigbo et al., 2008).

### Neutrality plot analysis

A neutrality plot (GC12 vs. GC3) can be employed to evaluate the balance between mutation and selection when codon bias is created. GC12 is the mean GC content in the codon’s first and second positions (GC1 and GC2), while GC3 is the GC content in the third position. A strong association between GC12 and GC3 implies that the species is primarily under mutation pressure; in contrast, a lack of correlation suggests that the main driving force is natural selection.

### Effective number of codons-GC3s plot analysis

The ENC-GC3s plot is a tool commonly used to determine whether the codon usage of a particular gene is affected by mutation alone or by other factors, such as natural selection. This plot consists of ENC values on the ordinate and GC3s values on the abscissa, with a predicted curve calculated by a specific formula. If the points are distributed around the expected curve, it can be concluded that mutation pressure is the primary factor in the formation of codon bias. However, if the points deviate significantly from the expected curve, it is likely that other factors, such as natural selection, are at play in the formation of codon bias.


$$ENC_{\exp}=2+GC3_{s}+\frac{29}{GC3_{s}^{2}+{\left(1-GC3_{s}\right)}^{2}}$$

The ENC_Ratio_ quantifies the divergence between the forecasted and actual ENCs.


$$ENC_{ratio}=\frac{ENC_{\exp}-ENC_{obs}}{ENC_{\exp}}$$

### Parity rule 2-bias plot analysis

Parity Rule 2 bias (PR2-bias) plot analysis can be used to identify the magnitude and direction of gene bias. This plot is based on A3/(A3+ U3) versus G3/(G3 + C3), and the center point of the plot indicates that the codon has no usage bias, with A = T and C = G.

### Correspondence analysis

Correspondence analysis (COA) is a widely accepted multivariate statistical analysis method used to recognize codon usage patterns. All genes were plotted in a 59-dimensional space, considering the 59 sense codons (Met and Trp excluded). This approach can detect the main trends in codon usage in the core CDSs of *Ganoderma* and arrange codons along the axis based on their RSCU value.

### Determination of optimal codons

The genes were arranged in descending order of their ENC values, and 10% of the genes from the beginning and end of the list were chosen to form a dataset of high- and low-expression genes. The difference in RSCU (ΔRSCU) between the two datasets was then calculated, with a ΔRSCU value of more than 0.08 indicating a codon with high expression. Codons with RSCU values higher than 1 were regarded as high-frequency codons. A codon with both ΔRSCU>0.08 and RSCU>1 was identified as an optimal codon.

### Phylogenetic analysis

Codon usage-based and mitochondrial sequence-based methods were applied to compare the phylogenetic relationships of *Ganoderma* strains. Using the RSCU values of the 13 *Ganoderma* strains, SPSS v19.0 software was used to apply a hierarchical clustering method to depict the relationships between the various *Ganoderma* strains as a tree. Utilizing the procedure documented in our earlier studies, we also constructed phylogenetic trees of the 13 *Ganoderma* strains based on the combined mitochondrial gene datasets (Li et al., 2019a, 2020b). The alignment of individual mitochondrial genes was first conducted through MAFFT v7.037 (Katoh et al., 2017), and then the aligned sequences were merged into one set with the use of Sequence Matrix v1.7.8 (Vaidya et al., 2011). A partition homogeneity test was applied to detect any potential inconsistencies in the phylogenies of different mitochondrial genes. Partition Finder 2.1.1 (Lanfear et al., 2017) was employed to ascertain the best model of partitioning and evolution for the combined mitochondrial gene set. The phylogenetic tree was constructed using MrBayes v3.2.6 based on the Bayesian inference (BI) method (Ronquist et al., 2012). Two independent runs, each with four chains (three heated and one cold), were conducted for a total of 2 million generations. Samples were taken every 1,000 generations, and the first 25% of these samples were discarded as burn-in. The remaining trees were used to calculate Bayesian posterior probabilities (BPPs) in a 50% majority-rule consensus tree.

## Results

### Nucleotide composition of *Ganoderma* core PCGs

The analysis of the codon usage of 12 core mitochondrial PCGs from 13 *Ganoderma* strains revealed that the average length of these genes ranged from 363 bp to 1983 bp, with *nad3* having the shortest average length and *nad5* having the longest. Out of these 12 core PCGs, 9 genes had varying sequence lengths among the different *Ganoderma* strains, while the *atp6*, *cox3* and *nad2* genes had the same gene length across all 13 strains. The *nad6* gene showed the greatest length variation, with a maximum difference of 180 bp. Furthermore, these 12 core PCGs was found to be rich in T bases, with an average content of 39.77%, followed by A bases, with an average content of 33.14%. The G and C base contents were relatively low, with averages of 14.14 and 12.96%, respectively. The average GC content of core PCGs ranged from 20.07 to 33.84%, with *rps3* having the lowest GC content and *cox1* having the highest. These results demonstrate that different *Ganoderma* strains display considerable differences in base composition and gene length, even within the same species, such as *G. lucidum* and *G. lingzhi*.

### Codon usage indicators

**Figure 1:**
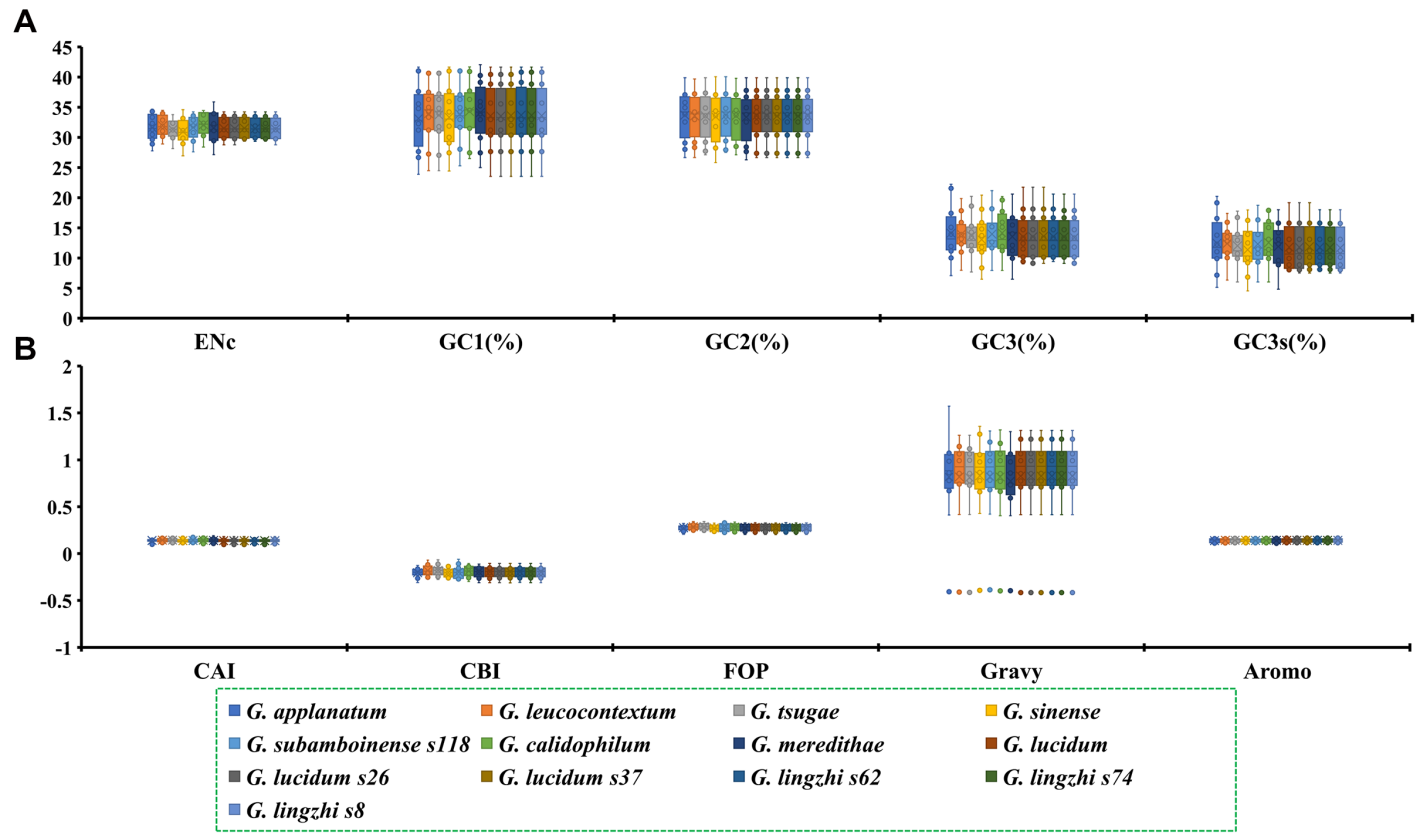
The codon usage indicators of twelve core protein-coding genes in 13 *Ganoderma* strains. **(A)** ENc, GC1, GC2, GC3, GC3s indicators. **(B)** CAI, CBI, FOP, Gravy, Aromo indicators.

The GC1, GC2 and GC3 contents of the 12 core PCGs in the 13 *Ganoderma* strains were 33.70, 33.55 and 13.65%, respectively (Figure 1). The average GC3s value of these 12 PCGs was 11.97%, indicating that the mitochondrial core PCGs of *Ganoderma* tend to terminate with an A or a T base. Furthermore, the indices of A3s, T3s, G3s, and C3s of the 12 core PCGs of the *Ganoderma* strains showed that the codons were more likely to end with A, followed by T, C and G, with values of 60.48, 47.00, 9.33, and 3.60%, respectively. An analysis of the codon bias of 12 core PCGs in the 13 *Ganoderma* strains was conducted. The CAI values of the core PCGs ranged from 0.099 to 0.182, with *nad2* having the lowest value and *cox2* having the highest. *G. lingzhi* s74 had the lowest CAI value, while *G. calidophilum* had the highest, demonstrating that they had a strong codon bias. The CBI values of the 13 *Ganoderma* strains ranged from −0.204 to −0.181, with *G. sinense* having the lowest value and *G. tsugae* having the highest. The core PCGs of the *Ganoderma* strains showed a range of CBI values from −0.297 to −0.107, with *nad2* having the lowest and *atp6* having the highest. The FOP values of the 12 core PCGs ranged from 0.213 to 0.327, with *nad2* having the lowest and *rps3* having the highest. *G. sinense* had the lowest FOP value, while *G. tsugae* had the largest. The GRAVY values of the 12 core PCGs were mostly positive, indicating that they were likely hydrophobic proteins, with the exception of *rps3*, which was considered hydrophilic. The AROMO values of the PCGs ranged from 0.110 to 0.168, with *cob* having the highest and *rps3* having the lowest. The GRAVY values of the 13 *Ganoderma* strains ranged from 0.774 to 0.831, with an average of 0.820. The three *G. lucidum* strains showed considerable variations in the ENC, GC3s, GC, and AROMO indicators, while the three *G. lingzhi* strains showed variations in CBI, FOP, ENC, GC3s, GC, GRAVY, and AROMO values, indicating that the frequency of synonymous codon usage also varied within *Ganoderma* species.

### Codon usage correlation analysis

**Figure 2:**
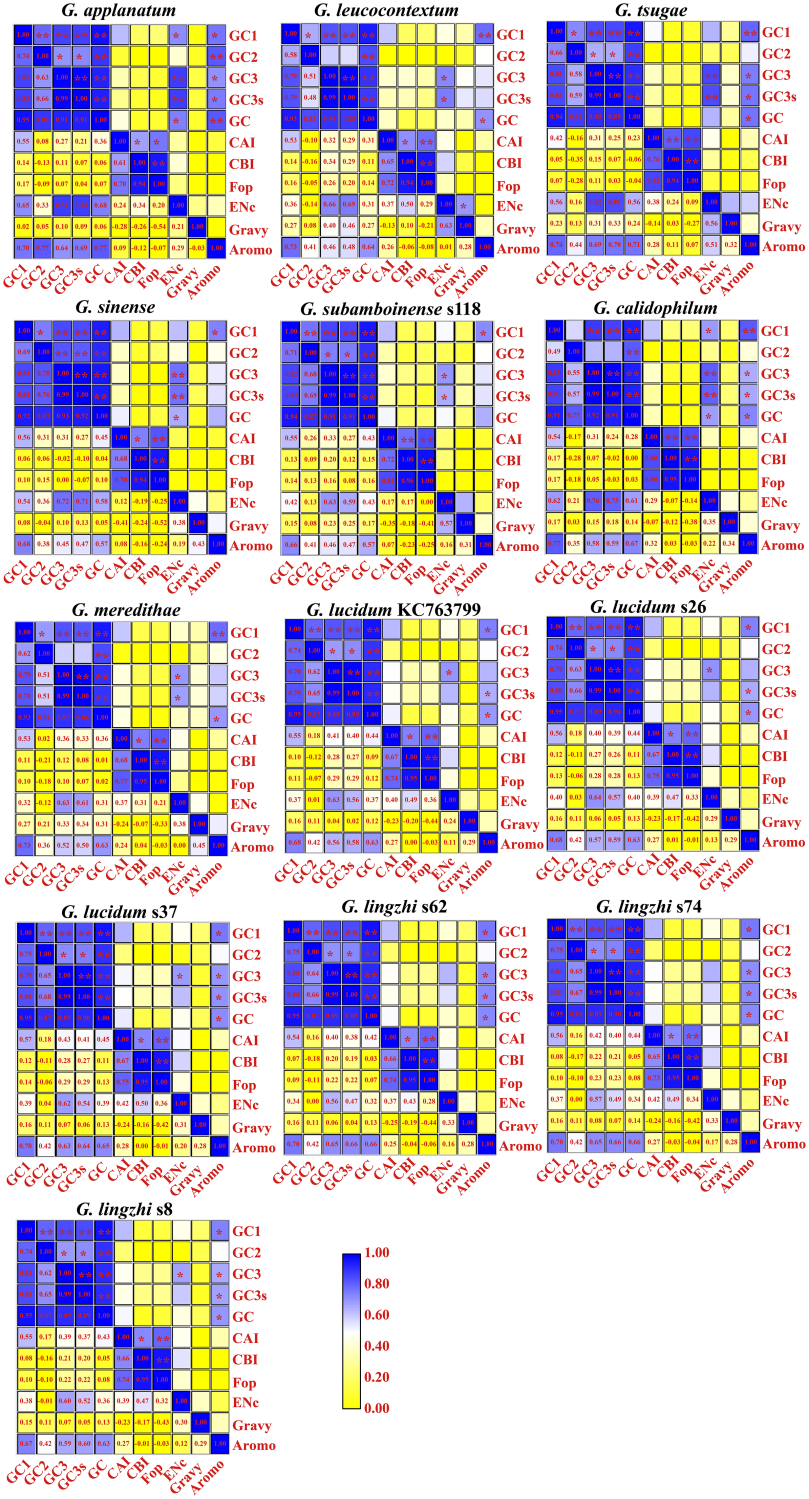
Pearson’s correlation analysis heatmap of codon usage indicators of 13 *Ganoderma* strains. The color of the blocks in the heatmap changes from yellow to blue, indicating an increase in the correlation coefficient. A single asterisk signifies a statistically significant correlation between the two (Continued)FIGURE 2 (Continued)indicators at the *p* < 0.05 level, while two asterisks symbolize a significant correlation between the two indicators at the *p* < 0.01 level. The 13 *Ganoderma* species are *G. applanatum*, *G. leucocontextum*, *G. tsugae*, *G. sinense*, *G. subamboinense* s118, *G. calidophilum*, *G. meredithae*, *G. lucidum* KC763799, *G. lucidum* s26, *G. lucidum* s37, *G. lingzhi* s62, *G. lingzhi* s74, and *G. lingzhi* s8, from left to right and from top to bottom.

A strong association was observed between the GC1 content of mitochondrial codons and GC3, GC3s, GC, and AROMO values in all 13 *Ganoderma* strains (*p* < 0.05; Figure 2). Additionally, a significant correlation was found between the GC2 content and GC content (*p* < 0.01). Moreover, the GC3 content was significantly correlated with GC3s and the GC content (*p* < 0.01), and it was also found to influence the ENC value in 11 out of the 13 *Ganoderma* strains, with the exceptions of *G. lingzhi* s62 and *G. lingzhi* s74. Additionally, the GC3s content was significantly associated with the ENC value in 7 *Ganoderma* strains (*p* < 0.05), with the exceptions of *G. lucidum* and *G. lingzhi*. Furthermore, the GC content was significantly correlated with the AROMO values in 12 out of the 13 *Ganoderma* strains (*p* < 0.05), with the exception of *G. sinense*. Moreover, the CAI of mitochondrial codons was significantly correlated with the CBI and FOP in all 13 *Ganoderma* strains (*p* < 0.05). A significant correlation was also observed between the CBI value and FOP value in all *Ganoderma* strains (*p* < 0.05).

### Neutrality plot analysis

**Figure 3:**
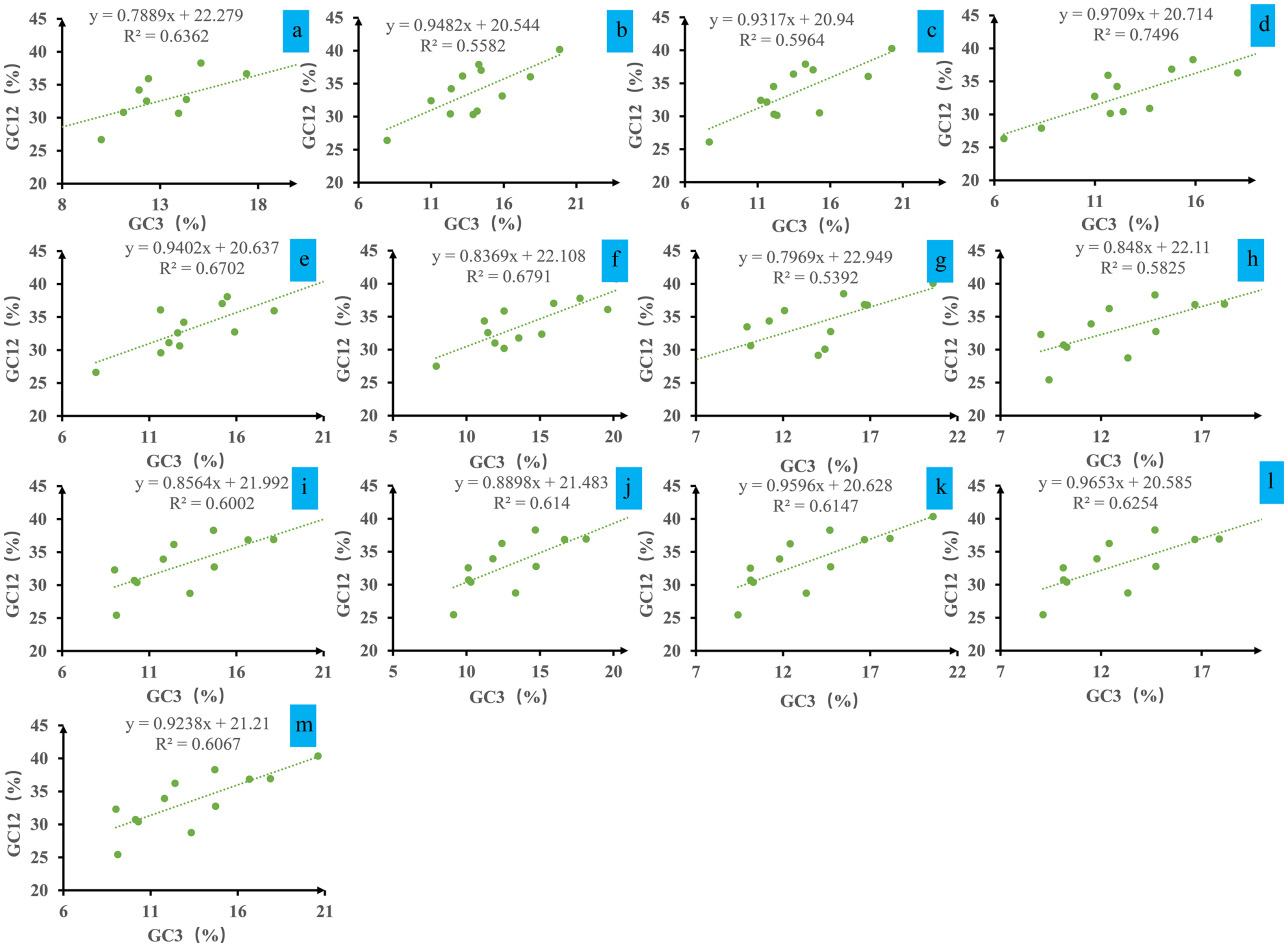
Neutrality plot analysis of GC12 and the third codon position (GC3) for the entire coding DNA sequence of 13 Ganoderma strains. **(A)** *G. applanatum*; **(B)** *G. leucocontextum*; **(C)** *G. tsugae*; **(D)** *G. sinense*; **(E)** *G. subamboinense* s118; **(F)** *G. calidophilum*; **(G)** *G. meredithae*; **(H)** *G. lucidum* KC763799; **(I)** *G. lucidum* s26; **(J)** *G. lucidum* s37; **(K)** *G. lingzhi* s62; **(L)** *G. lingzhi* s74; **(M)** *G. lingzhi* s8.

Analysis of the neutrality plot between the GC12 and GC3 contents in *Ganoderma* mitochondrial codons revealed a weak positive correlation, with regression coefficients ranging from 0.79 to 0.97 and R2 values ranging from 0.5392 to 0.7496 (Figure 3). The GC12 content varied from 25.44 to 40.26%, and the GC3 content varied from 6.47 to 20.60%. Data points deviated from the trend line. Statistical analysis indicated that there was no significant correlation between the two values (*p* > 0.05), indicating that natural selection was the major factor influencing codon bias in *Ganoderma*.

### Effective number of codons-GC3s plot analysis

**Figure 4:**
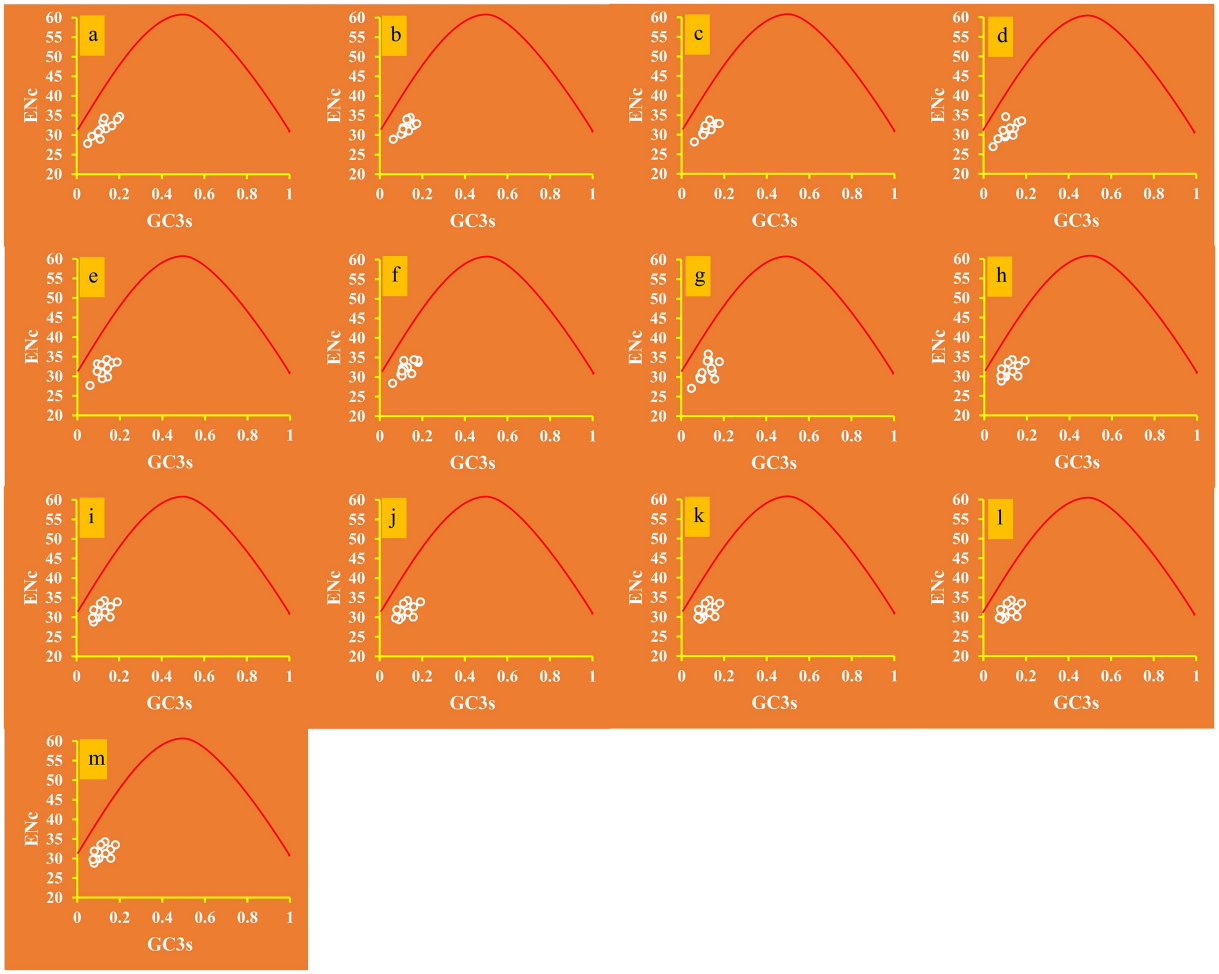
ENC-GC3 plot analysis of 12 core PCGs in 13 *Ganoderma* strains. The solid line represents the expected curve when codon usage bias is affected only by mutation pressure. **(A)** *G. applanatum*; **(B)** *G. leucocontextum*; **(C)** *G. tsugae*; **(D)** *G. sinense*; **(E)** *G. subamboinense* s118; **(F)** *G. calidophilum*; **(G)** *G. meredithae*; **(H)** *G. lucidum* KC763799; **(I)** *G. lucidum* s26; **(J)** *G. lucidum* s37; **(K)** *G. lingzhi* s62; **(L)** *G. lingzhi* s74; **(M)** *G. lingzhi* s8.

The average ENC value of the 12 core PCGs detected was 31.53, which is lower than 35, indicating a strong codon usage preference of proteins (Figure 1). The ENC values of the 13 *Ganoderma* strains ranged from 30.96 to 32.11, confirming the strong codon usage preference of *Ganoderma* species. The ENC plot showed that all *Ganoderma* genes were below the expected ENC-plot curve (Figure 4), suggesting that factors other than mutational pressure, such as natural selection, may be involved in codon bias formation.

The ENC_Ratio_ values for all core PCGs were found to be greater than the expected values (Supplementary Figure S1), ranging from 18.22 to 19.50%. This suggests that GC3s play an essential role in the formation of codon bias. Consequently, it can be deduced that natural selection is a major factor in the formation of *Ganoderma* codon bias.

### Parity rule 2-bias plot analysis

**Figure 5:**
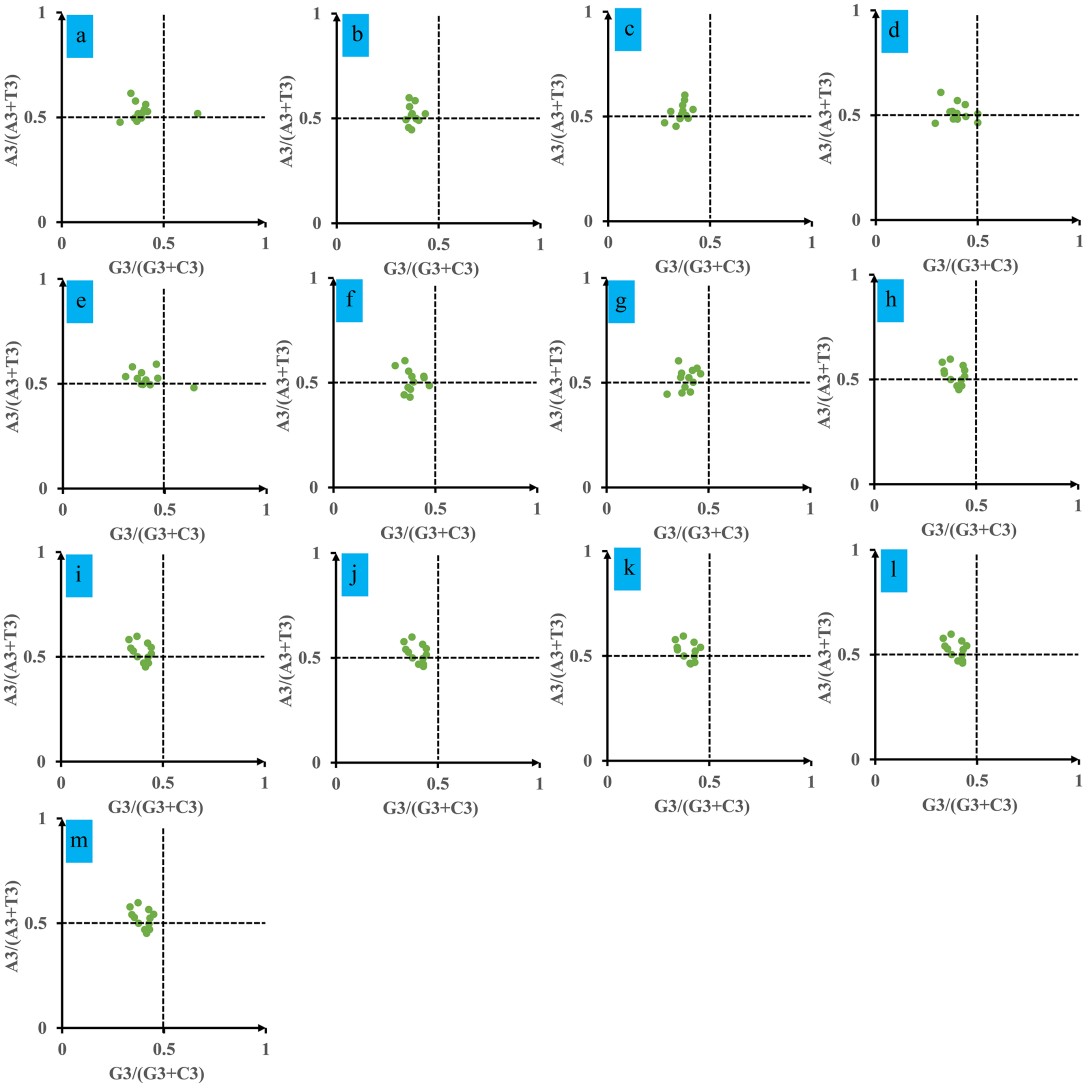
Parity Rule 2 (PR2) plot analysis of 12 core PCGs in 13 *Ganoderma* strains. **(A)** *G. applanatum*; **(B)** *G. leucocontextum*; **(C)** *G. tsugae*; **(D)** *G. sinense*; **(E)** *G. subamboinense* s118; **(F)** *G. calidophilum*; **(G)** *G. meredithae*; **(H)** *G. lucidum* KC763799; **(I)** *G. lucidum* s26; **(J)** *G. lucidum* s37; **(K)** *G. lingzhi* s62; **(L)** *G. lingzhi* s74; **(M)** *G. lingzhi* s8.

An analysis of Parity Rule 2 (PR2) plots was conducted to ascertain any biases in the mitochondrial genes of *Ganoderma* (Figure 5). The plot was centered on 0.5 and divided into four quadrants. The results indicated a strong preference for An over T and C over G at the third base of the mitochondrial codon of *Ganoderma*. Most of the dots were found in the fourth quadrant, followed by the third quadrant. With the exception of *G. applanatum* and *G. subamboinense*, the *Ganoderma* strains were not located in the first or second quadrant. This suggests a clear codon usage preference in *Ganoderma* species.

### Correspondence analysis

**Figure 6:**
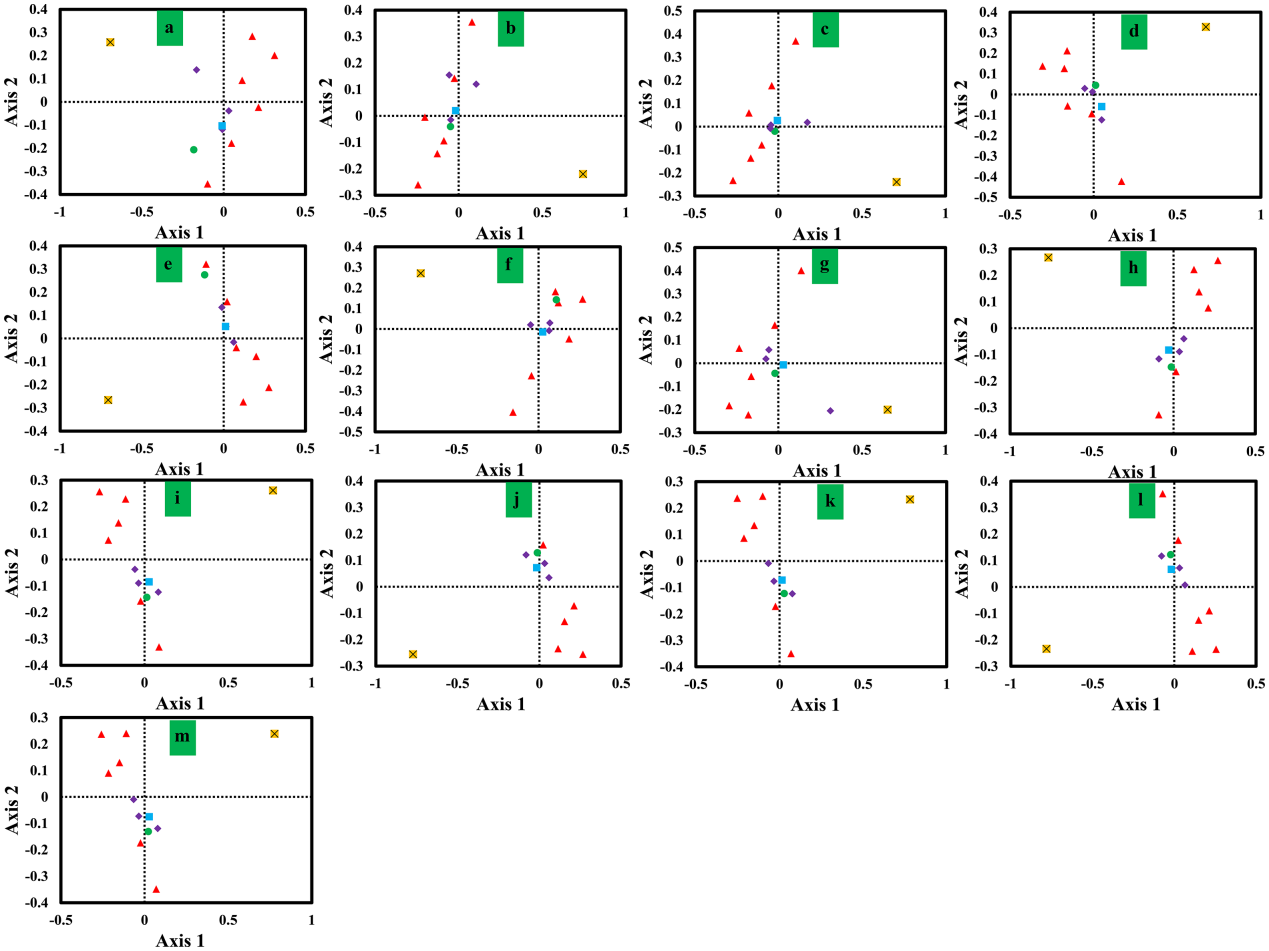
Correspondence analysis (COA) based on the relative synonymous codon usage (RSCU) values of 12 mitochondrial genes from 13 *Ganoderma* strains. Purple represents the *cox* gene, red represents the *nad* gene, green represents the *atp6* gene, blue represents the *cob* gene, and yellow represents the *rps3* gene. **(A)** *G. applanatum*; **(B)** *G. leucocontextum*; **(C)** *G. tsugae*; **(D)** *G. sinense*; **(E)** *G. subamboinense* s118; **(F)** *G. calidophilum*; **(G)** *G. meredithae*; **(H)** *G. lucidum* KC763799; **(I)** *G. lucidum* s26; **(J)** *G. lucidum* s37; **(K)** *G. lingzhi* s62; **(L)** *G. lingzhi* s74; **(M)** *G. lingzhi* s8.

We conducted a correspondence analysis (COA) based on the RSCU values of mitochondrial genes from 13 *Ganoderma* strains to further analyze codon biases (Figure 6). Axis 1, Axis 2, Axis 3 and Axis 4 were the main contributors to variance, with average contribution rates of 29.15, 17.00, 10.82 and 9.11%, respectively. Axis 1 had the highest contribution to variance. Pearson correlation analysis revealed a significant correlation between Axis 1 and GC3s, CAI, CBI, and ENC values. Moreover, we observed considerable variation in the *rps3* gene and other core PCGs, indicating the differentiation of synonymous codon usage of core PCGs.

### Optimal codon analysis

**Figure 7:**
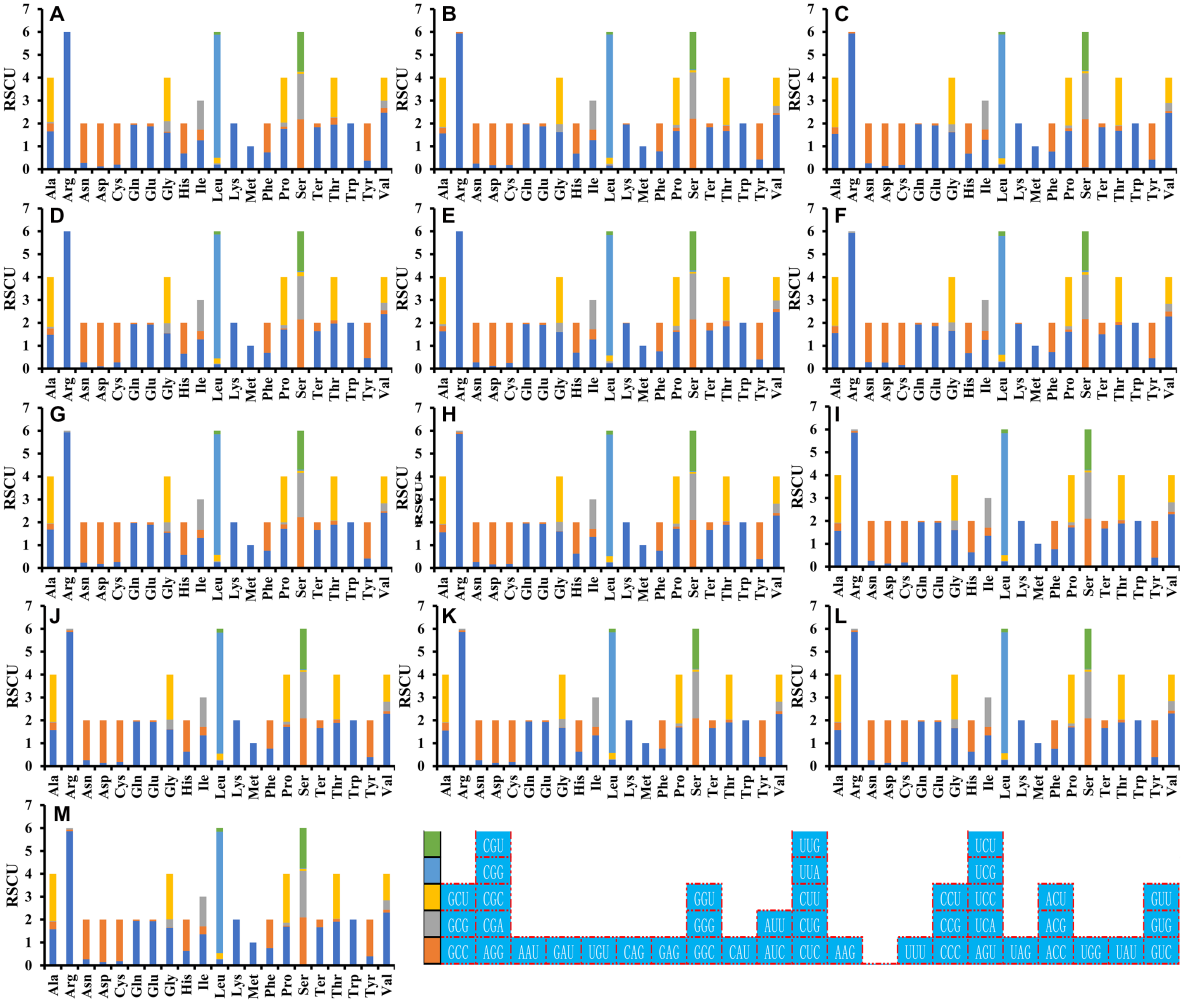
Relative synonymous codon usage (RSCU) analysis of 12 mitochondrial genes from 13 *Ganoderma* strains. **(A)** *G. applanatum*; **(B)** *G. leucocontextum*; **(C)** *G. tsugae*; **(D)** *G. sinense*; **(E)** *G. subamboinense* s118; **(F)** *G. calidophilum*; **(G)** *G. meredithae*; **(H)** *G. lucidum* KC763799; **(I)** *G. lucidum* s26; **(J)** *G. lucidum* s37; **(K)** *G. lingzhi* s62; **(L)** *G. lingzhi* s74; **(M)** *G. lingzhi* s8.

**Figure 8:**
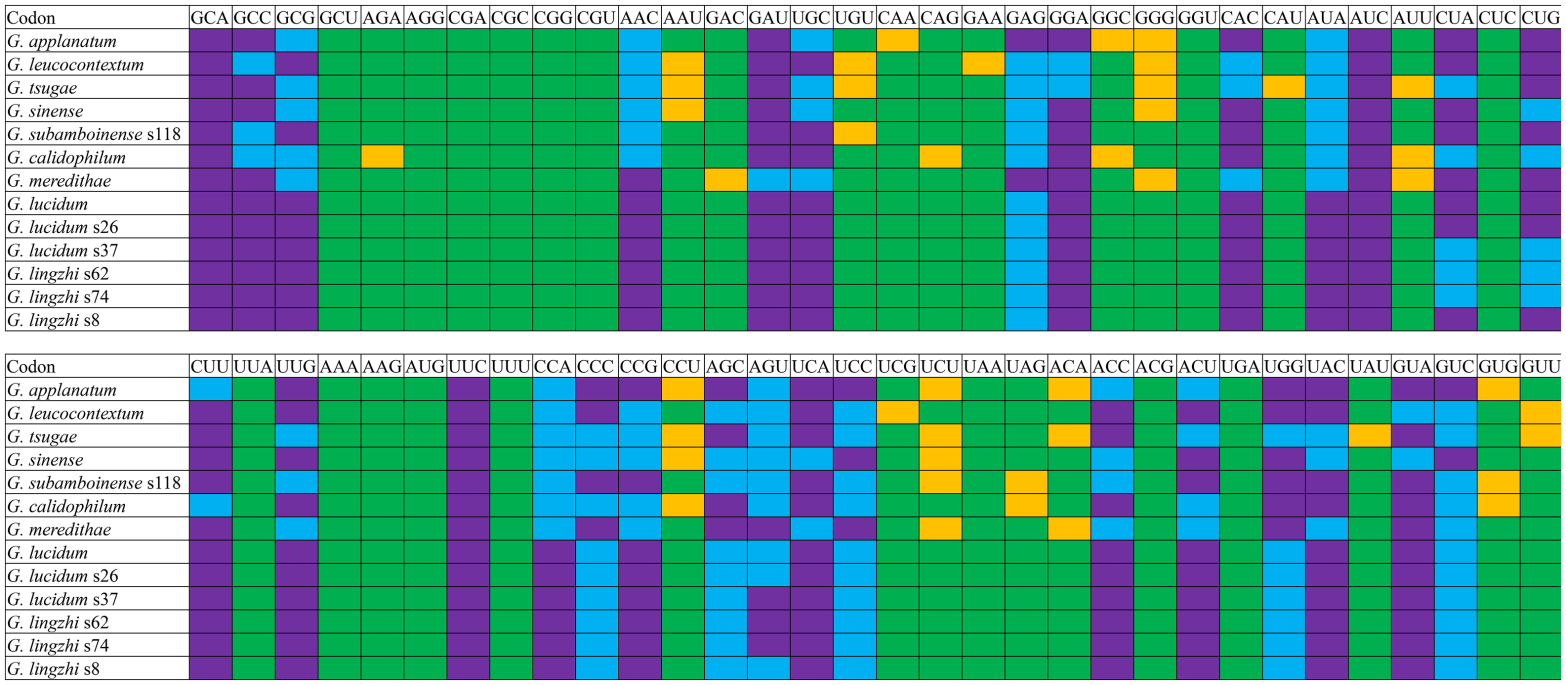
Optimal codon analysis of 13 *Ganoderma* strains (ΔRSCU>0.08 and RSCU>1) are marked in purple. Highly expressed codons (ΔRSCU>0.08) are marked in yellow, and high-frequency codons (RSCU>1) are marked in blue.

Analysis of the relative synonymous codon usage (RSCU) of the 13 *Ganoderma* strains revealed 29 high-frequency codons shared among the species (Figure 7). Of these, 12 ended in C, 7 in A, 6 in G, and only 4 in T, indicating a preference for high-frequency codons ending in C. Furthermore, 27, 22, 21, 18, 22, 21, 21, 22, 22, 21, 21, 21, and 22 highly expressed codons (ΔRSCU>0.08) were identified in the *Ganoderma* strains (Figure 8) *G. applanatum*, *G. leucocontextum*, *G. tsugae*, *G. sinense*, *G. subamboinense* s118, *G. calidophilum*, *G. meredithae*, *G. lucidum* KC763799, *G. lucidum* s26, *G. lucidum* s37, *G. lingzhi* s62, *G. lingzhi* s74, and *G. lingzhi* s8, respectively. *G. lucidum* and *G. lingzhi* exhibited differences in the number and types of highly expressed codons within the respective species. Comparative analysis revealed 20, 16, 11, 14, 18, 14, 16, 22, 22, 21, 21, 21, and 22 optimal codons (ΔRSCU>0.08 and RSCU>1) in *G. applanatum*, *G. leucocontextum*, *G. tsugae*, *G. sinense*, *G. subamboinense* s118, *G. calidophilum*, *G. meredithae*, *G. lucidum* KC763799, *G. lucidum* s26, *G. lucidum* s37, *G. lingzhi* s62, *G. lingzhi* s74, and *G. lingzhi* s8, respectively. GCA, AUC, and UUC were the most frequently used codons of all 13 strains, followed by GAU, which was used as the optimal codon for 12 strains. GAG and GUC were both used as the optimal codons for the two species. Significant differences in the use of optimal codons were observed within or between *G. lucidum* and *G. lingzhi* species. CUA and GUA were chosen as the optimal codons for *G. lucidum* KC763799, *G. lucidum* s26, and *G. lingzhi* s8, while AGU was the optimal codon for *G. lucidum* s37, *G. lingzhi* s62, and *G. lingzhi* s74.

### Phylogenetic analysis

**Figure 9:**
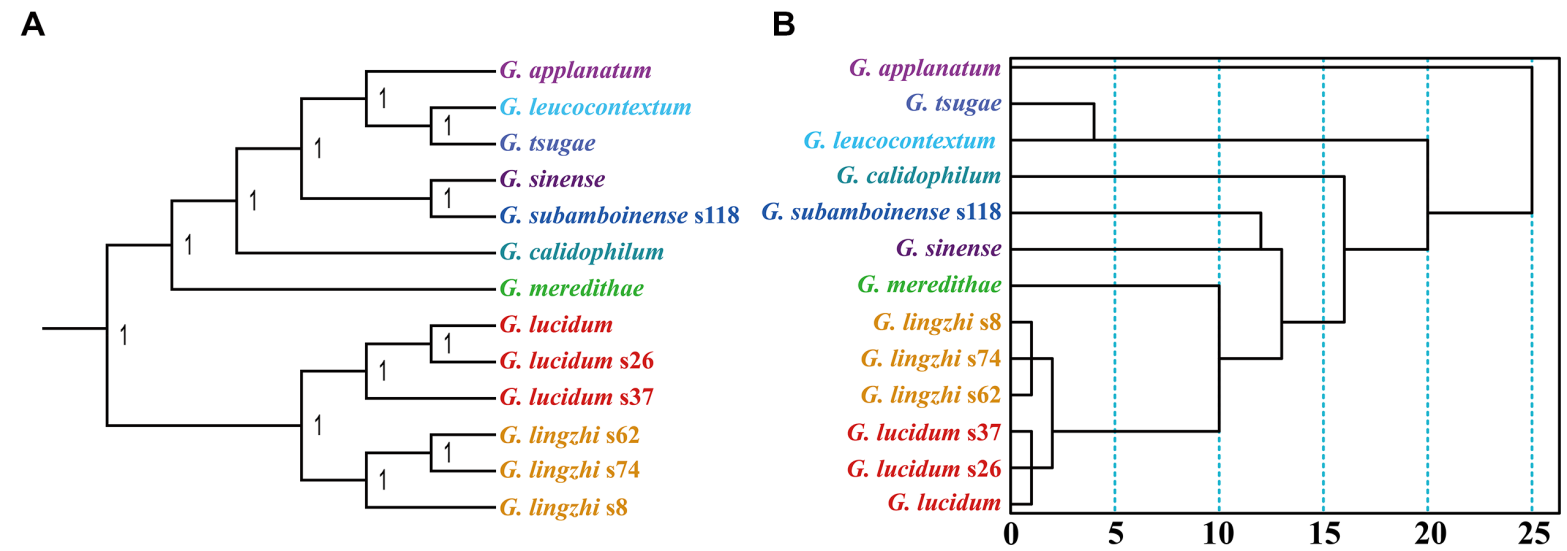
Relationship inference of 13 *Ganoderma* strains based on the Bayesian inference (BI) **(A)** method and relative synonymous codon usage (RSCU)-based hierarchical clustering **(B)**.

The Bayesian inference (BI) method was utilized to construct phylogenetic trees of 13 *Ganoderma* strains based on the combined mitochondrial gene set (Figure 9A). All the clades were well supported, with a BPP value of 1. The BI method identified *G. leucocontextum* as the sister species of *G. tsugae* and *G. sinense* as the sister species of *G. subamboinense*. Additionally, the six *G. lucidum* and *G. lingzhi* strains were grouped in the same evolutionary clade, demonstrating their close phylogenetic relationship. In contrast to the phylogenetic relationships inferred from sequences, those inferred from RSCU values had some discrepancies (Figure 9B), such as the phylogenetic status of *G. calidophilum*. Nevertheless, the RSCU-based tree also clearly revealed the close relationship between *G. leucocontextum* and *G. tsugae*, as well as between *G. sinense* and *G. subamboinense*. Furthermore, the relationships within *G. lucidum* and *G. lingzhi* were also clearly inferred using the RSCU method.

## Discussion

Advances in sequencing technology have allowed scientists to obtain the genetic sequences of a range of species and genome types, such as the nuclear genome, chloroplast genome and mitochondrial genome (Xu et al., 2015; Barbhuiya et al., 2020; Chakraborty et al., 2020; Tu et al., 2022). Genetic information analysis has revealed that the usage of synonymous codons varies between different species, with some codons being utilized more often than others (Liu et al., 2020). Mutations and selection are the most influential factors in codon usage bias; however, other elements, such as gene base composition, gene length, gene expression level, tRNA abundance, amino acid hydrophobicity, and aromaticity, also have an effect (Arella et al., 2021; Begum and Chakraborty, 2022; Liu et al., 2022). Analyzing the codon bias of various species can provide an understanding of the genetic structure and evolutionary trend of the species (Xing et al., 2020; Xiao et al., 2022). The *Ganoderma* genus encompasses numerous species, some of which are employed in traditional Chinese medicine and others characterized as plant-infecting fungi (Kües et al., 2015; Zhang et al., 2019). In recent times, the cultivation of edible and medicinal *Ganoderma* fungi has significantly increased in both economic and medicinal value (Lin, 2019; Li Z. et al., 2019). The exogenous synthesis of the medicinal components of *Ganoderma* has also become a major research focus (Kladar et al., 2016; Gong et al., 2019; Angulo-Sanchez et al., 2022; Bao et al., 2022). Examining the codon bias of *Ganoderma* species could further our comprehension of the evolution, genetics, and biosynthesis of active ingredients of *Ganoderma* species. However, the codon usage of organelle genomes of higher fungi, especially *Ganoderma* species, has not been extensively researched.

Mitochondria are commonly known as the auxiliary genome of eukaryotes (Li et al., 2020c, 2023c). This study found considerable variation in the length and base composition of the mitochondrial core PCGs of various *Ganoderma* strains, even within the same species, suggesting the differentiation of *Ganoderma* mitochondrial genes. The majority of the differences in codon usage were observed at the third codon. It was also noted that the core PCGs of *Ganoderma* species typically terminate with A/T, which is consistent with the mitochondrial codon usage pattern observed in some other eukaryotes (Mazumder et al., 2021; Montana-Lozano et al., 2022). Additionally, there was a disparity in base usage between different species and genes. *G. lucidum* and *G. lingzhi* exhibited changes in various base bias indicators, such as CAI, CBI, FOP, ENC, and GC3s values, suggesting that the frequency of base synonymous codon usage varied within *Ganoderma* species. The results also showed a correlation between codon base composition and GC3s, CAI, CBI, and FOP values, suggesting that base composition has an impact on codon bias. A low ENC value (below 35) is indicative of a strong codon preference (Prabha et al., 2017; Pepe and Keersmaecker, 2020). The mitochondrial core PCGs of *Ganoderma* had an average ENC value of 31.53, which is lower than 35 and thus demonstrates a strong codon preference. The disparity between the expected and actual ENC values was considerable, with a range of 18.22 to 19.50%. The neutrality plot and PR2-bias plot analyzes also provided evidence that natural selection plays a role in the codon bias of *Ganoderma*, which is consistent with the results from the mitochondrial genomes of other species (Barbhuiya et al., 2021a,b). The findings of this study revealed that despite some discrepancies in codon usage indicators between or within *Ganoderma* species, all of the fungi experienced strong natural selection on their mitochondrial PCGs.

It is speculated that the ancestors of eukaryotes obtained mitochondria from bacteria (Lang et al., 1999), and most mitochondrial genes have been shifted to the nucleus (Adams and Palmer, 2003). Many eukaryotes still possess some core PCGs, tRNA genes and rRNA genes for energy production (Bullerwell et al., 2000; Costa et al., 2012), which can be employed as molecular indicators for phylogenetic analysis. The mitochondrial genome is seen as a beneficial asset for establishing the phylogenetic relationships between species (Li et al., 2015; Johri et al., 2019). A combined mitochondrial gene set was employed to analyze the genetic relationships between various *Ganoderma* species and strains, and the results demonstrated a high level of support for each evolutionary clade. The relative synonymous codon usage (RSCU) of a range of *Ganoderma* species and strains was evaluated to identify relationships between them, which diverged from those established from sequence-based data. The RSCU value also accurately reflected the genetic connections between or within some *Ganoderma* strains or species, which was consistent with the findings of previous studies (Crane et al., 2021; Gupta et al., 2021; Li et al., 2022d). In general, this research has improved our understanding of codon usage characteristics and genetic evolution of this higher fungal group and other related fungal species. Furthermore, it has been demonstrated that phylogenetic results based on RSCU values can be a useful supplement to those based on sequences.

## Data availability statement

The datasets presented in this study can be found in online repositories. The names of the repository/repositories and accession number (s) can be found in the article/Supplementary material.

## Author contributions

QL, PW, and MG: conceived and designed experiments. YL, AS, WX, ZX, JH, and XC: analyzed the data. PW and QL: wrote and reviewed the manuscript. MG: project management. All authors contributed to the article and approved the submitted version.

## Funding

This study is supported by the Natural Science Foundation of Sichuan Province (no. 2023NSFSC1229) and Key R&D Project of Sichuan Provincial Department of Science and Technology (2023YFN0062).

## Conflict of interest

The authors declare that the research was conducted in the absence of any commercial or financial relationships that could be construed as a potential conflict of interest.

## Publisher’s note

All claims expressed in this article are solely those of the authors and do not necessarily represent those of their affiliated organizations, or those of the publisher, the editors and the reviewers. Any product that may be evaluated in this article, or claim that may be made by its manufacturer, is not guaranteed or endorsed by the publisher.
