# Supplementary material for: Comprehensive analysis of codon bias in 13 Ganoderma mitochondrial genomes
Source: Front Microbiol. 2023 May 4;14:1170790. doi: 10.3389/fmicb.2023.1170790 (PMC10192751; doi:10.3389/fmicb.2023.1170790)
Supplement: Supplementary file 1 [file Image_1.pdf]

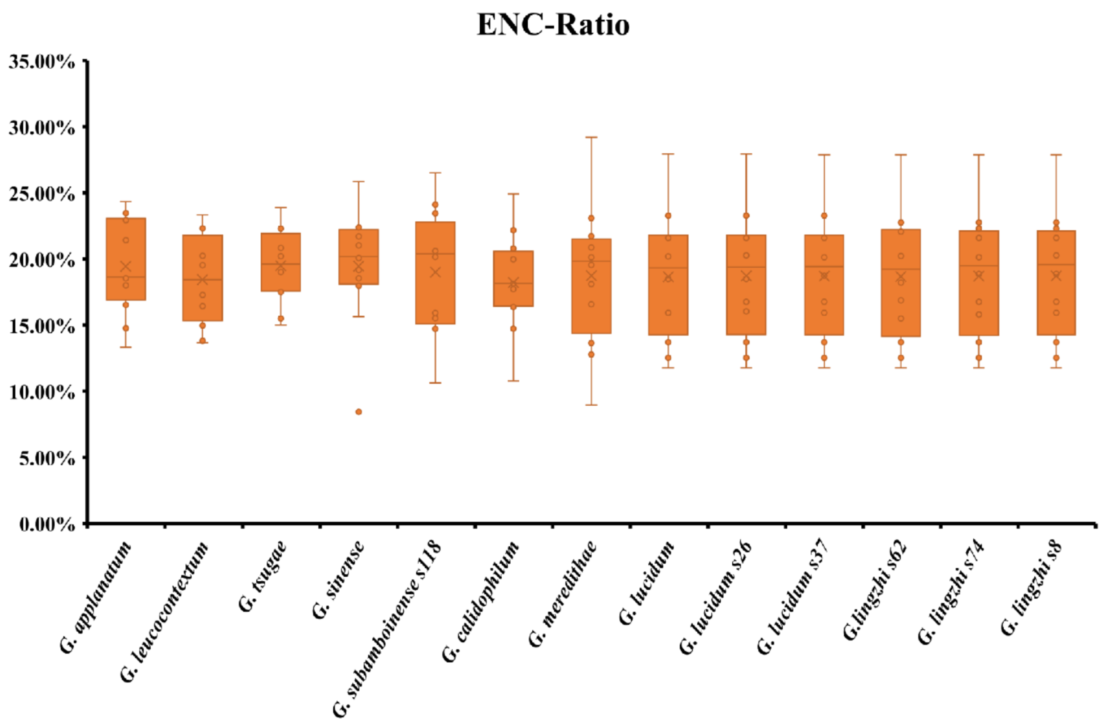

Figure S1 Variability of expected and actual ENC values of 12 mitochondrial genes from 13 *Ganoderma* strains.
